# Decreasing Health Disparities for People with Disabilities through Improved Communication Strategies and Awareness

**DOI:** 10.3390/ijerph120303301

**Published:** 2015-03-19

**Authors:** Nancy Sharby, Katharine Martire, Maura D. Iversen

**Affiliations:** 1 Department of Physical Therapy, Movement and Rehabilitation Sciences, Bouve College of Health Sciences, Northeastern University, Boston, MA 02115, USA; E-Mails: n.sharby@neu.edu (N.S.); Martire.k@husky.neu.edu (K.M.); 2 Department of Women’s and Children’s Health, Karolinska Institute, Stockholm, 171 76, Sweden; 3 Section of Clinical Sciences, Division of Rheumatology, Immunology and Allergy, Brigham and Women’s Hospital, Boston, MA 02115, USA; 4 Department of Medicine, Harvard Medical School, Boston, MA 02115, USA

**Keywords:** health disparities, barriers, communication, strategies

## Abstract

Factors influencing access to health care among people with disabilities (PWD) include: attitudes of health care providers and the public, physical barriers, miscommunication, income level, ethnic/minority status, insurance coverage, and lack of information tailored to PWD. Reducing health care disparities in a population with complex needs requires implementation at the primary, secondary and tertiary levels. This review article discusses common barriers to health care access from the patient and provider perspective, particularly focusing on communication barriers and how to address and ameliorate them. Articles utilized in this review were published from 2005 to present in MEDLINE and CINAHL and written in English that focused on people with disabilities. Topics searched for in the literature include: disparities and health outcomes, health care dissatisfaction, patient-provider communication and access issues. Ineffective communication has significant impacts for PWD. They frequently believe that providers are not interested in, or sensitive to their particular needs and are less likely to seek care or to follow up with recommendations. Various strategies for successful improvement of health outcomes for PWD were identified including changing the way health care professionals are educated regarding disabilities, improving access to health care services, and enhancing the capacity for patient centered care.

## 1. Introduction

People with disabilities (PWD) make up a large percentage of the United States population. Census data from 2012 informs us that 18.7% of the civilian, non-institutionalized population, or 56.7 million people have a disability [1]. Of those individuals, 38.6 million have a severe disability. They are also the poorest, most under educated and most vulnerable members of society. Approximately 14.7% of PWD between the ages of 16 and 64 who are interested in being employed are unemployed, compared to 7.2% of those without disabilities [2]. Many PWD earn less than $25,000 per year and 19% of those who have a severe disability live in poverty [3]. In addition to these disproportionate levels of unemployment and poverty, PWD are the most costly consumers of U.S. healthcare services [3,4].

The concept of “disability” covers a broad range of impairments, including sensory, mobility, behavioral and learning disorders. Further, these conditions usually occur along a spectrum so that two people with the same impairment, such as loss of vision, may have very different levels of function, and need varying levels of care and support, while others may have additional co-occurring illnesses. Disability crosses all ethnic and racial lines, as well as gender, age and socioeconomic status, creating greater individual differences. While it is clear that the health care needs of PWD are complex, many health care providers do not take into account how their different lived experiences impact access to care when designing services. Further, the additional time and interventions needed have not been successfully negotiated with health care plans and regulatory policies to provide adequate funding for these modifications [5].

### 1.1. Who is Disabled?

To begin a discussion of health disparities and disabilities, it is important to first consider, who is disabled? The traditional definition of disability is based on a medical model that equates disability with an impairment of one or more body functions or structures that interferes with activities. In other words, an impairment that impacts performance is equivalent to having a disability. The 1990 Americans with Disabilities Act (ADA) expands this concept by defining someone with a disability as a person who has a physical or mental impairment that substantially limits one or more major life activities [6]. The ADA definition includes people who had an impairment, even if they do not currently have one, and those who are perceived by others as having a disability [6]. Impairment, or disability, is viewed as a negative health outcome and health professionals have traditionally perceived their task as preventing, treating, or curing such outcomes. The etiology of disability is perceived to lie solely within the client and the only way to improve function is for a health care professional to eliminate, remediate or repair the problem. Thus, the medical model is reductionist and is often described as a deficit model.

**Figure 1:**
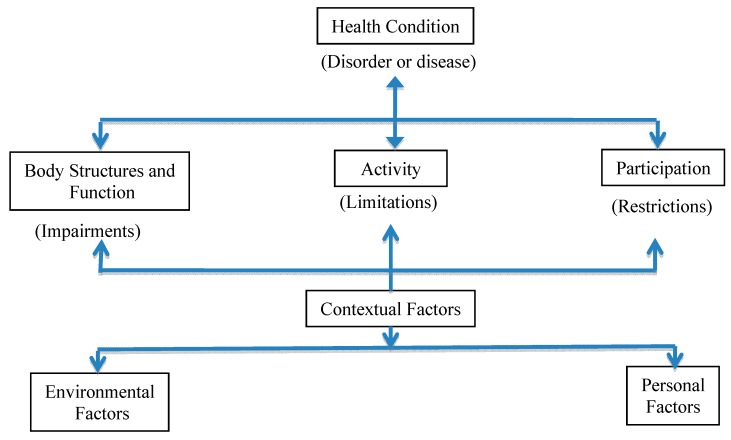
International Classification of Function, Disability and Health (ICF) (WHO 2001) (http://www.who.int/classifications/icf/en/), adapted from [9].

However, newer approaches to conceptualizing disability include social and integrative models. In these paradigms, impairment alone does not drive disability, and participation, not functional capacity, is the desired goal [7]. Social models of disability emphasize environmental barriers arising from negative attitudes such as stigma and oppression, and physical barriers as the most important factors that cause disability. Unlike the medical model that places impairment as the driver of disablement, the social model stresses the importance of access and social accommodations to facilitate participation [7]. Advocates for the social model stress the need for change in attitudes of people without disabilities and modification of physical and social environments to maximize access and increase inclusion. Under the social model, an impairment such as lower extremity paralysis would not be considered the cause of a disability. Rather, this model considers the inaccessibility of the built environment to be an important cause [8]. For example, an individual may have the best wheelchair available, but if there are no curb cuts, ramps or elevators than he/she would be unable to access the environment and participate in activities which are important to him/her. Further, he/she may be unable to gain employment due to the negative stigma surrounding disability. While it is important to ameliorate impairments when possible, many PWD are able to participate in activities that are important to them and to have a high quality of life as a result of environmental modifications. In the medical model, disability is considered a tragedy, while in the social model, disability is viewed as an aspect of diversity [9]. However, neither model can sufficiently explain the disablement process. Thus, newer integrative models have been developed. Most notable, is the *International Classification of Functioning, Disability and Health* (ICF) (Figure 1) developed by the World Health Organization (WHO) [10].

In 2001, the WHO approved the ICF as a method to provide a uniformed system to universally describe and measure disability. It is an ecological model where all factors contributing to disability are equally important and interact with all other factors, ultimately leading to the client’s ability, or inability, to participate in activities that are important to him/her. The ICF uses disability as an umbrella term that considers all factors that impact function: diagnosis, impairments and participation restrictions such as work and social roles [10]. One important component of this model is the identification of biological factors such as the diagnosis or disease process, and the impact on body functions or structures that cause impairments *(i.e.*, visual impairment, intellectual impairment, mobility impairment). For example, arthritis (a health condition) may cause joint inflammation, pain, stiffness, and tissue degeneration (change in body function or structure). These impairments may lead to activity limitations such as difficulty opening a jar, walking, or climbing stairs. However, the ICF differs from the medical model through its emphasis on participation in important life activities rather than the achievement of specific tasks. If someone is unable to walk through the grocery store (activity), he/she may still be able to shop independently (participation) by using a small scooter provided by many stores. The provision of the free scooter in the store is an example of modifying an environmental factor. Thus, *participation* is not synonymous with activity. Factors that enable participation are referred to as contextual factors. In the environment, these factors may include a physical environment that is free of architectural barriers, and a social network that values positive attributes more than deficiencies. Personal factors that impact capacity may include education, personality, age, motivation and job skills.

Another important policy document that integrates medical and social models of disability developed by the WHO, is the Convention on the Rights of People with Disabilities (CRPD) [11]. This convention was adopted in 2006 and provides a blueprint for the rights of PWD internationally that is based on the ADA but is broader in scope. The CRPD defines disability very explicitly as an evolving concept and states, “disability results from the interaction between persons with impairments and attitudinal and environmental barriers that hinders full and effective participation in society on an equal basis with others”.

The ICF model does not equate impairment alone with disability, because disability arises from a complex and fluid interaction of biological, social/environmental and personal factors [10]. However, most health care providers have been educated to conceptualize disability in terms of the medical model and focus their efforts on deficit reduction. This thinking can be positive because it allows for treatments and interventions that can minimize or correct impairments. However, this thinking may be at odds with how PWD view themselves and what they want from their providers. The attitudes the health professional brings to the health encounter will provide a subtle but important background to relationship building and shared decision making. Many clients believe their interests and needs are unheard because medical providers view them as disabled people rather than people with impairments who lead different types of lives. They may feel de-valued, and disrespected [12]. While subtle, these differences in attitudes may lead the health providers to focus too heavily on the impairment and not what the client is seeking: care for an acute illness not related to the disability, referral for durable medical equipment, or advice about sexually related concerns such as birth control. These differences can create communication barriers that lead to lack of adherence to medical plans and diminished outcomes. They may also be a factor in the development of secondary conditions.

### 1.2. Health Disparities and Persons with Disabilities

“Health care disparities refer to differences in health status or healthcare which are considered both avoidable and unfair” [13]. A more global definition of health disparities describes disparities as differences in health access and outcomes between specific populations and the general population. In 2009, the Disability Policy Consortium reported that PWD in Massachusetts rate their health as poor more often than people without disabilities [14]. For example, PWD rate their health as excellent or very good only half as often as people with no disabilities, and rank their health as fair or poor four times more often. Further, PWD experience a 4-fold risk of developing diabetes and they are three times more likely to commit suicide. There is a higher prevalence of overweight and obesity among PWD compared to the general population. In addition, they are 2 to 4 times more likely to be substance abusers [14].

Jones *et al.* [15] enrolled a sample of 258,279 adults in a study designed to explore the interplay of mobility impairments and racial/ethnic minority status on health disparities They reported that heart problems, breathing problems, low back pain, joint symptoms, cancer, and hearing impairments were most frequently identified in people with mobility impairment who were not a racial/ethnic minority. Diabetes, hypertension, stroke and visual impairments were highest among people with mobility impairments who were members of a racial/ethnic minority. Non-disabled people, not from racial/ethnic minorities, had the lowest rate of obesity, followed by people without mobility impairment who came from racial ethnic minorities. People with mobility impairments alone and those who are minority and had mobility impairment had the highest rates of obesity [15]. This study highlights disparities in both physical and mental health that occur in people with mobility impairments, which are increased with minority group status. These data increase our understanding of the complexity of providing health care for PWD who are at the intersection of two under-served populations.

Satisfaction with health care is lower in PWD. They are ten times more likely than non-disabled people to report low satisfaction with their health care. Further, the more serious the disability, the less satisfied PWD are with their health care [16]. Adults with complex activity limitations *vs.* those with non-complex or no activity limitations were more likely to have had a clinical encounter in the last twelve months where the health provider did not listen carefully to them, explain things in a way they could understand, show respect for what they had to say or spend enough time listening to them.

PWD often require more, and different, interventions and accommodations to receive adequate health care [5]. Despite the increasing population of PWD, one California survey [17] demonstrated that only 3.6% of primary care facilities had a scale that was accessible for wheelchairs and only 8.4% had adjustable exam tables. Modified equipment is not readily available in many medical sites. Further, even if adaptive equipment is available, it is often not properly used and staff may use unsafe measures to transfer or move patients. PWD have described having an entire physical exam performed in their wheelchair. Unfair treatment, often resulting from assumptions and stigmas by physicians, contributes to PWD not wanting to follow up with their health care [5]. These inadequacies have led to misdiagnoses, fewer preventative measures and ultimately fewer care visits by PWD who are less likely to return to a provider who cannot safely or effectively examine them. In addition, PWD are offered fewer appointments for preventative measures including flu shots, gynecological examinations, and mammograms leading to an increased risk of preventable diseases.

Multiple factors contribute to overall poor health in PWD that are not secondary to the disability itself including lack of financial resources [2]. With lower incomes, there is often not enough money for health care after paying bills for necessities such as rent, food and utilities. In cases of diseases such as cancer, the strength of treatments can be the difference between remission and death [5]. Though medical staff attempt to modify their treatments, many times their efforts can be insulting, and do more harm than good. One woman reported that during a routine procedure, radiotherapy staff were unable to completely secure her arm with Velcro straps. Instead, they used masking tape to secure her upper limb to the table to be able to get adequate pictures [3]. The woman felt that the staff had done what was easiest for them, and had neglected her own comfort.

Thus, PWD experience health care delivery in a different manner than those without a disability. These experiences can lead to disparities in health care and poorer health outcomes. This literature review aims to synthesize the literature on factors impacting health care disparities among PWD, paying close attention to physical access and communication barriers, and how to address and ameliorate them. These factors are easily modifiable at an individual level and have the potential for great impact. However, to be more readily enforced, changes in policy may also be necessary.

## 2. Methods

For this review, the term “disability” was limited to impairments that impact function and participation in life activities due to movement and mobility limitations, sensory impairments, or psychiatric conditions. Search terms used were: “people with disability/disabilities”, “disabilities and health care satisfaction”, PWD and satisfaction “communication with health care professionals”, “health care communication”, “disability and disparities” and “disability and medical education”. The following databases were searched for relevant articles: PubMed (primarily the subset MEDLINE), and CINAHL. Articles written in English and published between 1 January 2005 and 1 November 2014 were included. Citations of selected articles were also used to find other applicable articles. Only full text articles available online were searched. Severity of disability was not an inclusion or exclusion criteria. Articles representing the array of study designs and reviews were included such as qualitative assessments, systematic reviews, narrative reviews, cohort or longitudinal studies. Papers for this review were excluded if they were: commentaries, opinion pieces or perspectives, took place outside the United States, focused on only one racial/ethnic group, studied only one type of patient group (*i.e.*, Multiple Sclerosis) or studied a patient group known to have communication difficulties (*i.e.*, people with CVA, autism, dementia, intellectual delays), and studies which did not directly examine patient-provider communication or relationships with PWD as they related to health disparities.

Two reviewers conducted the primary screening of articles using a standard format. Initially, only titles of the articles were read to determine if they were applicable. Next, abstracts of selected articles were read. Abstracts that did not meet the inclusion criteria were noted and removed. Articles meeting the inclusion criteria were acquired. A third level screening occurred after reading the full text of these articles. A list of article titles was kept throughout the search process to help eliminate duplicate publications.

### Data Extraction and Analysis

The articles identified were a mix of qualitative studies, mixed methods studies, cross-sectional surveys and quasi-experimental studies. Thus, a formal quality ranking score for articles could not be conducted. Rather data from studies were organized into categories or themes and presented in a summative manner.

## 3. Results and Discussion

**Figure 2:**
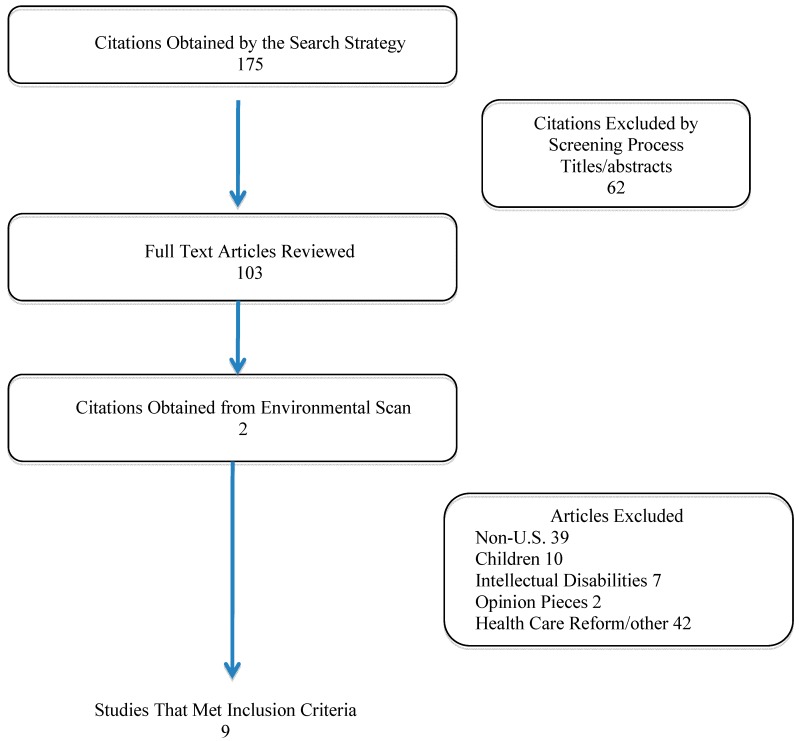
Results of literature search stratified by category.

Nine articles met the inclusion criteria [17,18,19,20,21,22,23,24,25] (Figure 2). Of these, three involved focus groups, three were survey studies and three were quasi-experimental studies. Of the articles identified, four studies focused on medical education and medical students’ perceptions and experiences [18,19,20,21], two focused on access to care (physical barriers) [17,22], one study examined providers’ experiences with PWD [23], one reported psychiatric PWD’s experiences with medical care [24] and one assessed both providers’ and patients’ experiences and perceptions of the care of PWD [25]. Based on these nine studies, the researchers identified the following themes: medical students’ knowledge and attitudes regarding PWD, provider perceptions and patient perceptions of health care delivery and health outcomes, and access barriers. From these articles, the team synthesized strategies that may be used to enhance health care delivery and diminish disparities in PWD.

### 3.1. Medical Students’ Knowledge and Attitudes

Research has shown that health professionals and health care students are often unsure how to interact with and treat PWD. A focus group study by Iezzoni [18] revealed that medical students admitted knowing little about PWD, but were open to changing their views. The students surveyed admitted to having discriminatory attitudes towards morbidly obese people who may be considered disabled due to their limited mobility status. They also reported not knowing whether or not it was appropriate to talk to their patients about the disability and having little knowledge about disabilities in general. Further, many students reported negative views regarding PWD because their point of reference was typically older family members who had acquired impairments. On a positive note, many students recognized they needed greater training. In contrast, when the students’ school administrations were surveyed about their students’ competency, administrators felt their students graduated with enough skill to treat all patients. These findings are reinforced by the results of another study that assessed medical student’s examination skills in an objective structured clinical examination (OSCE) [19]. Students demonstrated significantly more difficulty in both inter-personal skills and physical examination skills when the patient had a disability, indicating the need for more training in this area.

A study was designed to examine medical residents’ attitudes and/or knowledge about PWD before and after a short educational intervention. First year medical residents in the field of Physical Medicine and Rehabilitation (PM&R) and a control group of second and third year residents who did not receive the training were enrolled [20]. Participants were surveyed about their knowledge and attitudes toward PWD at three time points: prior to training, immediately after the training, and three months later. The seven-hour training consisted of two hours of factual information, followed by presentations by PWD and family members, role playing and group discussions. Trainee scores rose significantly on the test for attitudes toward PWD and were maintained by the 3-month follow up. Although the initial attitude scores of the trainee group were lower than the controls pre-training, it was higher after the training. It is important to note that even among physicians who choose to work with PWD and their families, explicit training in humanistic aspects of disability is useful.

Symons *et al.* [21] conducted a study to examine the impact of integrating a series of educational experiences into the four-year curriculum at an American medical school. Learning experiences included both direct instruction and structured interactions with PWD, their caregivers, and professionals who care for them. Fourth year residents had the opportunity to participate in an additional four-week clerkship that provided primary care to PWD. This study compared students’ attitudes and comfort level working with PWD to medical students enrolled in a program without the disability enhanced curriculum. Surveys were completed prior to the beginning of medical school training and at the end of the third year. The results of the academic intervention moved students in a positive direction with changes being at or near significance. See Table 1.

**Table 1:** Studies addressing quality of health care delivery and disparities in persons with disabilities (PWD).

Authors (Year)	Design and Sample	Intervention or Major Aim	Outcomes
*Medical Education and Medical Student Perceptions, Attitudes and Comfort Level Working with PWD*			
Symons *et al*, 2014 [21]	Non-randomized controlled study Rx: Medical students enrolled in public medical school with disparities curriculum C: Medical school students from similar institution	**Rx:** Disabilities curriculum integrated across all four years of study—includes lectures on disability and society, communication with PWD, small group encounters with PWD, precepted clerkship in clinic for PWD, 1/2 workshop on legal and socioeconomic context of caring for PWD, and potential 4-week elective on primary care for PWD	Students in the intervention group reported better attitudes and higher comfort level working with PWD in the following areas: greater comfort working with PWD when someone else is with them (*p* = 0.008); more positive attitudes towards PWD and perceptions of PWD (*p* = 0.001). However, male students in the Rx group who encountered PWD in a clinical context had a tendency to agree with more negative statements about PWD
*Medical Education and Medical Student Perceptions, Attitudes and Comfort Level Working with PWD*			
Brown *et al*, 2010 [19]	Quasi-experimental 146 3rd year medical students engaged in family medicine clerkship	Students engaged in 1 of 3 standardized patient (SP) experiences (1) patient without disability (n = 63); (2) patient with spinal cord injury (SCI) (n = 40); (3) patient with intellectual disability (ID) and his/her caregiver (n = 53)	Students involved in the OSCE with patients with SCI scored lower on history taking, physical exam, ordering of lab tests and interpersonal skills. Ordering of hemoglobin was higher among patients who did not have a disability (OR = 4.16; 95% CI = 1.78–9.17), ordering urinalysis was 3 times higher (OR = 3.08; 95% CI = 1.34–7.08) and oviding lifestyle counseling was 2 times higher (OR = 2.15; 95% CI = 1.04–4.44)
Moroz *et al*, 2010 [20]	Quasi- experimental Rx: 11 PMR residents C: 10 psychiatry residents engaged in standard	Rx: 7-h one day training including didactic lectures, panel presentations covering: disability facts; stories of experiences with medical care from PWD; information and skills on medical evaluation of disability. Following these didactic experiences students were assigned to a play the role of a PWD in a wheelchair or as a caretaker in structured simulations and debriefing sessions. C: Standard medical training	Students demonstrated significant improvements in disability knowledge and more positive attitudes towards PWD. Knowledge in sensitivity training did not persist at 3 months but positive attitudes toward PWD did.
Iezzoni *et al*, 2005 [18]	Focus group study Medical students during final year of study	Focus groups lasted 2 h	Students reported negative views of living with a disability, expressed admiration for PWD who are coping well, most drew their perceptions of PWD from family experiences, students voiced negative attitudes towards a subgroup of PWD, those who are obese and reported morbidly obese patients are responsible for their health status. Students also reported taking short cuts to save time and deal with busy schedules but did not realize this may impact their interactions with PWD.
*Patient and Provider Perceptions of Health Care and Outcomes*			
O’Day *et al*, 2005 [24]	Focus group 16 patients with psychiatric disabilities in a psychiatric rehabilitation program	Focus group lasted two hours to examine patient perceived barriers to care	PWD reported trouble finding a primary care physician with good communication skills, receiving inadequate information about medication side effects, lack of understanding of their health condition, excess costs due to inadequate health insurance.
*Patient and Provider Perceptions of Health Care and Outcomes*			
Bachman *et al* , 2006 [23]	Cross-sectional survey 379 health care providers from managed care organizations	No intervention	Providers more likely to provide care to patients with chronic illnesses, mobility, cognitive or psychiatric disabilities than those with communication disorders or visual impairments. Providers reported those with communication disorders are the most difficult to medically manage. The majority perceived PWD do not have easy access to medical care.
Morrison *et al*, 2008 [25]	Focus groups 27 health professionals and 19 adults with disabilities	Focus groups of PWD and providers	Both groups reported primary care providers need more education about PWD, improved education regarding communication and interpersonal skills, improved physical access at clinical sites, more flexible and accessible schedules for medical appointments.
Mudrick *et al*, 2011 [17]	Cross-sectional survey of provider sites conducted by nurses employed by different state health plans	No intervention	Barriers for PWD included physical barriers in bathrooms, examination tables, parking access, and access to buildings. 3.6% had an accessible weight scale and 8.4% had height accessible exam tables.
Lagu *et al*, 2013 [22]	Cross-sectional survey of 256 endocrinology, gynecology, orthopedic surgery, dermatology, urology, ophthalmology, otolaryngology, and psychiatry practices	Researchers posed as a fictional patient who was obese and had hemiparesis, used a wheelchair and could not transfer without assist	56 (22%) practices reported they could not accommodate the patient, 9 (4%) buildings were inaccessible, 47 (18%) reported they could not transfer the patient to an exam table and 22 (9%) had height adjustable tables or lifts for transfer. Of all practices, gynecology offices were the least accessible

Rx = treatment group; C = control group; OSCE = Objective structured clinical exam; PMR = Physical medicine and rehabilitation.

### 3.2. Physician and Patient Perceptions

Physicians often believe that the only barriers to their provision of services to PWD are access related and often do not consider attitude or knowledge issues [25]. Several studies have examined this broader array of barriers. In Massachusetts, researchers mailed surveys to all providers practicing in two large networks whose services occurred at multiple types of facilities. Providers reported that the most common barriers to care for PWD were “lack of insurance, transportation, difficulty making appointments, difficulty communicating needs to providers, and difficulty understanding staff” [23] (p. 133). Physical access issues were also reported.

In a series of focus groups, Morrison *et al* [25] explored the experiences of PWD when receiving medical care and the experiences of the primary health care providers treating these adults. PWD identified three concerns: lack of preventative care, financial barriers, and dissatisfaction with the care they received. The primary complaint about the care provided was the lack of physical access. In addition, they perceived primary care providers lacked the necessary time, clinical training, equipment, and resources to provide adequate care for their complex medical needs [25]. The patients provided a number of specific concerns regarding their care, such as discomfort or negative attitudes, lack of availability of important services at the office, untrained staff, and lack of provider skill or knowledge. Patients further reported negative provider attitudes and misinformation that led to misdiagnoses. Providers described feeling uncomfortable working with PWD. Many providers felt PWD should receive care in specialized settings. Interestingly, the provider focus groups reported the same barriers and issues as the clients. The combination of patients’ fear of inadequate treatment and the professionals’ beliefs that they are not prepared to provide care, helps explain why people with disabilities receive less preventative treatment and are less satisfied with the care they do receive.

Over half of PWD report difficulty concentrating and understanding what their doctor is saying [24]. This barrier may arise in patients with psychiatric disorders, intellectual disabilities (ID), hearing impairments and communication disorders. These are concerns expressed by many patients, but patients with psychiatric illnesses experience some particular challenges. Psychiatric conditions are prevalent, affecting one in five Americans. Although some of these individuals, who will have only mild symptoms, can be easily managed [26], there is significant stigma towards this population even among those working in the health professions. This stigma can be explained by the long misunderstanding of these conditions and the challenging symptoms some people with mental illness display. Ongoing research consistently demonstrates that this population has significantly higher rates of poor health. For example, 80% of these individuals present with at least one co-occurring condition and are more likely to be overweight or obese. Many patient-related factors lead to poor medical care and outcomes in this subgroup such as impaired communication skills, difficulty negotiating a complex medical system, difficulty trusting medical professionals and feelings of powerlessness. However, providers also contribute to disparity in care by dismissing the physical symptoms of medical illness that bring the patient with mental illness to the primary care doctor [24]. As with other disabling conditions, health care financing and lack of adequate insurance are also important.

A focus group study conducted by O’Day [24] revealed that according to patients, communication with health care providers was the most important factor affecting good care. As has been identified by other groups of PWD, patients want their providers to respect them as individuals, provide information about complex medical procedures and to trust that PWD are capable of accurately reporting symptoms. Patients with disabilities reported a unique problem affecting communication between PWD and health care providers was the health professionals’ attitudes toward PWD, and an appropriate attitude was identified as essential for a good relationship. Another perceived area of concern among PWD was physicians’ lack of knowledge about psychiatric illnesses and treatments, especially regarding medications and their side effects.

### 3.3. Access Barriers

Physical access issues have also been recognized as significant barriers to participation in appropriate medical care [17]. In a recent study, California medical reviewers conducted on-site reviews of 2389 primary care offices using a 55-item physical accessibility survey to determine accessibility to care [17]. The researchers reported few sites were totally physically and structurally accessible. When compared to prior research that relied on provider self-reports, the findings of the study illustrated much lower rates of physical access then had previously been reported. In addition to limited accommodations within medical offices, many buildings lacked adequate handicapped parking, curb cuts, ramps, railings and other necessities. While access to primary care is challenging, access to sub-specialty care is even more limited [22]. A telephone survey was performed to determine access issues in the offices of 256 subspecialty practices in four U.S. cities. In this study, a caller who purported to be someone with a mobility impairment, who was obese and could neither ambulate nor transfer to an examination table, contacted the medical office to make an appointment. Twenty-six percent of practices indicated they could not accommodate the patient, and gynecology practices had the highest rate of access barriers with 44% stating they could not see the patient. As stated above, these data are unacceptable given the fact that medical providers are also legally obligated to provide care and access to PWD under Title II (private entities) and III (public accommodations) of the ADA.

### 3.4. Strategies to Improve Access to Health and Health Outcomes in PWD

In general, PWD do not feel health care professionals are adequately prepared to treat them, and do not possess the skills to effectively communicate and develop trusting relationships with PWD [25]. In addition, many feel that their health professionals have negative attitudes and incorrect assumptions about PWD. For example, they may believe that their care is compromised due to their providers’ lack competence and knowledge about their disability. Similarly, many students in health profession programs reported a lack of adequate training leading them to feel less competent when working specifically with PWD. Patients report this issue can be resolved in multiple ways, including making primary care offices physically accessible, ensuring the provider skill level competence and sufficient time to address their needs [17].

In multiple studies [3,24,25] patients have consistently stated that they want to be heard and to have their providers learn from them. Iezzoni [12] offers two simple but effective strategies for overcoming patient-provider communication barriers. They are based on the provision of patient-centered care that emphasizes respect for patients and patients’ preferences, needs, and values [12]. This requires open communication where physicians are interested in learning the clients’ goals, aspirations and abilities, rather than relying on what they believe these to be. The first strategy is to make no assumptions about what clients want. Health care providers should not assume that they know what a patient wants or what type of health care they would like to receive. Many times patients are the experts on their own conditions, having lived with symptoms and side effects of medications, thus knowing their own needs quite well. This is particularly so in patients who have rare or uncommon conditions. Patients are also the experts on what they would like the outcome to be. The second strategy is to have no pre-conceived ideas about what is important to clients. In this paradigm, physicians should simply ask clients to describe their needs and the outcomes they are seeking. Others support this point of view and explain the importance of communicating to patients and not relying on the input of others [25].

Medical education programs are required to teach students about cultural competence and how to work with patients that have different cultural, ethical, and religious backgrounds. However, few programs offer their students the opportunity to specifically learn about disabilities, even though there is evidence that such efforts can be successful [27,28]. For example, a group of medical students were asked to write down words they felt described PWD before and after a four day training course on disabilities. Prior to the training, a majority of the students used de-personalized or negative words. However, when surveyed afterwards, students were significantly more empathetic and held more positive views [29]. It is well-known that medical schools have an enormous amount of material which must be taught, but not enough time is spent on disabilities in general and on intellectual disabilities in particular [30]. Since almost one in five non-institutionalized Americans identify as disabled, an increased emphasis on these topics during medical training would provide physicians the competencies needed to treat all their patients. Further, medical students need to know about the physical nature of disabling conditions and need to be taught the human dimension of living with a disability. It is important to note that many PWD rate their quality of life much higher than their medical providers. In fact, their perceived quality of life parallels the self-perceived quality of life of people without disabilities. Therefore, many PWD do not want to be “fixed”, but would like to receive health care that allows them to make autonomous decisions. They want to be treated with the same dignity and respect that would be afforded to patients without impairment.

Communicating with others is a learned skill and health professionals in training need access to opportunities to learn this skill by working with PWD under the guidance of a mentor [31]. There are also explicit skills that need to be acquired such as how to make eye contact with a person in a wheelchair, learning to speak directly to the patient even when an interpreter is present, and how to interview patients who have speech or communication difficulties. Finally, Kirschner [31] has identified six core competencies she believes should be included in medical education regarding PWD. These cover the areas of theoretical constructs of disability, physical assessment skills, etiquette, legal and statutory issues and patient centered care.

Many authors identify time constraints as a barrier to good communication about complex medical problems. [3,23,31] Lack of time also impedes the possibility of developing a trusting, respectful relationship. There are significant financial constraints imposed by third party re-imbursement that must be dealt with a policy level in order to change these funding barriers. While it is beyond the scope of this article to specifically address these policy issues, advocacy efforts are needed to develop healthcare systems that can be both cost effective and allow extra time for visits that are required by PWD. Perhaps this increased time can be justified as an intervention that can promote patient-centered care, provide a better understanding of the patient’s needs and wishes in order to increase health care satisfaction and health outcome improvements. Addressing both physical and time-related access issues will require that providers become knowledgeable about legal and policy issues such as the ADA and the Affordable Health Care Act. Both of these policies require PWD to have complete access to physical spaces and health care resources [31]. Table 2 provides strategies for health care providers to decrease health disparities for PWD.

**Table 2:** Strategies to decrease health disparities for PWD.

Recommendations for Health Care Providers to Help Decrease Health Disparities for PWD
Make no assumptions about what PWD want.
Ask PWD what their preferences are for treatment interventions.
Acquire knowledge about conditions that cause disability, functional impacts of these conditions and effective interventions.
Develop physical assessment skills to properly examine PWD.
Develop sensitivity to the disability experience.
Engage in patient-centered care with all health encounters.
Be knowledgeable about state and federal statutes that govern accessibility for PWD such as the Americans with Disabilities Act and the Affordable Care Act.
Create accessible treatment spaces, including parking and signage.
Advocate with third party payers and others to provide adequate time and resources for an effective client encounter.

## 4. Conclusions

There are multiple access barriers to appropriate health care for PWD. Too often PWD find that medical providers do not demonstrate respectful attitudes toward them and make inappropriate assumptions about their needs. Lack of sufficient time for appointments, physical inaccessibility, cost of care and transportation present additional barriers. Patients may defer necessary appointments because of past experiences where they felt disrespected by providers who did not seem to care about their input, and whom they feared may not have put enough value on their lives. This review reinforces the significance of effective communication between patients and clinicians that is dependent on the practice of patient-centered care [32]. The more satisfied patients are with their health care and the more comfortable they are with practitioners, the more likely they are to attend follow-up appointments and utilize preventative measures. Improved patient-provider communication is an important way to create improved overall health outcomes and patient satisfaction, thus decreasing the disparities that exist between disabled and non-disabled persons. Additionally, health care providers need to be educated about their legal as well as moral obligation to provide physical access to care for PWD. To ensure compliance with ADA, medical and other health professional curricula should emphasize the legal obligations to provide access to care for PWD.
